# Transcriptional convergence after repeated duplication of an amino acid transporter gene leads to the independent emergence of the black husk/pericarp trait in barley and rice

**DOI:** 10.1111/pbi.14264

**Published:** 2023-12-20

**Authors:** Bo Li, Yong Jia, Le Xu, Shuo Zhang, Zhoukai Long, Rong Wang, Ying Guo, Wenying Zhang, Chunhai Jiao, Chengdao Li, Yanhao Xu

**Affiliations:** ^1^ Hubei Key Laboratory of Food Crop Germplasm and Genetic Improvement & Key Laboratory of Ministry of Agriculture and Rural Affairs for Crop Molecular Breeding, Food Crops Institute Hubei Academy of Agricultural Sciences Wuhan China; ^2^ Western Crop Genetics Alliance, Future Food Institute, Western Australian State Agricultural Biotechnology Centre, College of Science, Health, Engineering and Education Murdoch University Murdoch Western Australia Australia; ^3^ Hubei Collaborative Innovation Centre for the industrialization of Major Grain Crops, College of Agriculture Yangtze University Jingzhou China; ^4^ Department of Primary Industries and Regional Development South Perth Western Australia Australia

**Keywords:** barley, black husk/pericarp, *Blp*, melanin, convergent evolution, rice

## Abstract

The repeated emergence of the same trait (convergent evolution) in distinct species is an interesting phenomenon and manifests visibly the power of natural selection. The underlying genetic mechanisms have important implications to understand how the genome evolves under environmental challenges. In cereal crops, both rice and barley can develop black‐coloured husk/pericarp due to melanin accumulation. However, it is unclear if this trait shares a common origin. Here, we fine‐mapped the barley *HvBlp* gene controlling the black husk/pericarp trait and confirmed its function by gene silencing. The result was further supported by a yellow husk/pericarp mutant with deletion of the *HvBlp* gene, derived from gamma ray radiation of the wild‐type W1. *HvBlp* encodes a putative tyrosine transporter homologous to the black husk gene *OsBh4* in rice. Surprisingly, synteny and phylogenetic analyses showed that *HvBlp* and *OsBh4* belonged to different lineages resulted from dispersed and tandem duplications, respectively, suggesting that the black husk/pericarp trait has emerged independently. The dispersed duplication (dated at 21.23 MYA) yielding *HvBlp* occurred exclusively in the common ancestor of *Triticeae*. *HvBlp* and *OsBh4* displayed converged transcription in husk/pericarp tissues, contributing to the black husk/pericarp trait. Further transcriptome and metabolome data identified critical candidate genes and metabolites related to melanin production in barley. Taken together, our study described a compelling case of convergent evolution resulted from transcriptional convergence after repeated gene duplication, providing valuable genetic insights into phenotypic evolution. The identification of the black husk/pericarp genes in barley also has great potential in breeding for stress‐resilient varieties with higher nutritional values.

## Introduction

Convergent evolution, termed by some biologists as the ‘replaying the tape of life’ event, is a fascinating phenomenon whereby the same trait emerged repeatedly in distantly related organisms (Orgogozo, 2015; Washburn *et al*., 2016). Notable instances of phenotypic convergence include the independent evolution of echolocation in bats and whales (Shen *et al*., 2012), wings in birds and bats (Ben‐Hamo *et al*., 2016), camera‐like eyes in cephalopods and vertebrates (Fernald, 2006) and drug resistance in pathogens (Farhat *et al*., 2013). In plants, convergence evolution is also widespread [summarized by (Trewavas, 2014)] and has led to the repeated evolution of the C4 photosynthesis system (Heyduk *et al*., 2019), flower scents (Knudsen and Tollsten, 1995), nitrogen fixation (Trewavas, 2014), lignin biosynthesis (Weng *et al*., 2010) and lateral root organ (Naramoto *et al*., 2019). Most phenotypic convergences are believed to have a natural selection cause, i.e. the same phenotype only evolves in different species to address similar environmental challenges (Xu *et al*., 2020). For example, the C4 photosynthesis system in plants has evolved more than 60 times as an adaptation to drought and high‐temperature stresses (Heyduk *et al*., 2019). Other prominent cases of phenotypic convergences in plants have been related to adaptation to seasonal environmental change (Hu *et al*., 2003), specific pollinators (Knudsen and Tollsten, 1995), heavy metal toxicity (Preite *et al*., 2019; Ryan and Delhaize, 2010) and terrestrial environments (Naramoto *et al*., 2019). Compared to the widespread divergent evolution, phenotypic convergence occurs much less frequently and is often more challenging to identify because it requires the reconstruction of phenotypes along the phylogeny to prove their independent origins (Arbuckle *et al*., 2014).

The fundamental goal concerning convergent evolution studies is to uncover the molecular basis underpinning the observed phenotypic convergence. This would help answer an important question in evolutionary biology: how likely will the same ecological challenges result in similar phenotypic and genetic changes? (Trewavas, 2014). Theoretically, convergent phenotypes can arise from similar or different genetic changes depending on several factors: similar natural selection, phylogenetic history, population demography and genetic constraints (Rosenblum *et al*., 2014). Various types of genomic convergence such as amino acid substitution and gene expression have been reported and related to plant's adaption to extreme environments (Christin *et al*., 2010; Jia *et al*., 2020; Xu *et al*., 2020). Although these genomic convergences have improved our understanding of the genetic basis of convergent evolution, there are also clear limitations: firstly, most of these genomic convergences correspond to genetic mutations in the same or direct orthologous genes. Thus, the observed convergence can be generally attributed to genetic constraint effects. It remains unclear how phenotypic convergence may evolve independently from different genes; secondly, most associations between genomic convergence and phenotype adaptation were based on simple functional assumptions and lack empirical validation. Notably, these challenges also apply to other cases of phenotypic convergence currently reported in plants: metal toxicity tolerance in *Arabidopsis* (Preite *et al*., 2019), seed colour in common bean (McClean *et al*., 2018), the transition of outcrossing to selfing (Zhang *et al*., 2022), perenniality in rice and sorghum (Hu *et al*., 2003) and the development of plant shoot lateral organs (Naramoto *et al*., 2019). The limited cases of convergence evolution indicate that our understanding of plant phenotypic evolution remains fragmentary.

Grain colour of cereal crops is one of the most important agronomic traits subjected to both human and natural selections, which have been shown to significantly influence grain quality, yield and also environmental adaptability (Alemu *et al*., 2020; Bellido and Beta, 2009; Jia *et al*., 2020; Lang *et al*., 2021; McClean *et al*., 2018). In nature, mature grains of rice, maize, wheat and barley plants have evolved different colours such as yellow, purple, red, blue and black due to different pigmentations (Abdel‐Aal *et al*., 2006; Paulaneyer *et al*., 2017), potentially as a result of environmental adaptation because common pigments such as anthocyanins and flavonoids are strong antioxidants and can help plants address various stress conditions (Jia *et al*., 2020; Kaur *et al*., 2022; Shomali *et al*., 2022). Unlike other colours, the black grain trait, prominently observed in wild rice (Zhu *et al*., 2011) and wild barley (Long *et al*., 2019; Shoeva *et al*., 2020), was attributed to the accumulation of plant melanins instead of anthocyanins in the lemma and/or pericarp (Fei *et al*., 2021; Shoeva *et al*., 2020; Varga *et al*., 2016). Noteworthy, the black hull trait in rice needs to be differentiated from the deep purple colouration trait (often mistaken as black), which is mainly due to anthocyanin accumulation and is controlled by candidate genes in the anthocyanin biosynthesis pathway (Mackon *et al*., 2021; Oikawa *et al*., 2015; Upadhyaya *et al*., 2021). Black‐coloured rice and barley have attracted enormous human attention not only for their distinctive appearance but also due to their exceptional nutritional values as healthy foods (Ge *et al*., 2021; Shen *et al*., 2016). The accumulation of melanins, usually accompanied by enriched phenolics and flavonoids, exhibits strong antioxidant activity with proven beneficial effects on human health (Ge *et al*., 2021; Glagoleva *et al*., 2022). In addition to the antioxidant effects, the accumulation of melanins is also believed to increase the mechanical strength of seeds, protecting them from insect and mechanical damage (Jocković *et al*., 2020), and microbial pathogen infections (Choo et al., 2015; Glagoleva et al., 2020).

Due to its complicated metabolic nature, the genetic basis of melanin production in barley and rice has not been fully elaborated yet. To date, only 2 candidate genes *OsBh4* and *Phr1*, encoding a putative tyrosine amino acid transporter and a polyphenol oxidase, respectively, have been cloned in rice responsible for the black hull trait (Fukuda *et al*., 2012; Zhu *et al*., 2011). In barley, the black grain trait has long been known to be controlled by a single dominant locus *Blp* on chromosome 1H (Costa *et al*., 2001). Several previous studies have attempted to identify the *Blp* gene (Bungartz *et al*., 2016; Glagoleva *et al*., 2017, 2022; Jia *et al*., 2017; Long *et al*., 2019; Shoeva *et al*., 2016) and have narrowed it to an extremely small genetic region, containing only 21 annotated genes (Long *et al*., 2019). However, the corresponding candidate gene is still hiding in mystery. In this study, we overcome this challenge by screening a large collection of inbred lines pinpointed the *Blp* gene and confirmed its function by gene silencing. We found that the black husk/pericarp trait in barley was caused by gene insertion variation. Synteny and phylogenetic analyses suggested that *HvBlp* and *OsBh4* have evolved from different lineages, implying an independent origin. We also proposed a potential regulatory network for *HvBlp* and melanin biosynthesis in barley.

## Results

### *HvBlp* encodes an amino acid transporter homologous to rice *OsBh4*

**Figure 1:**
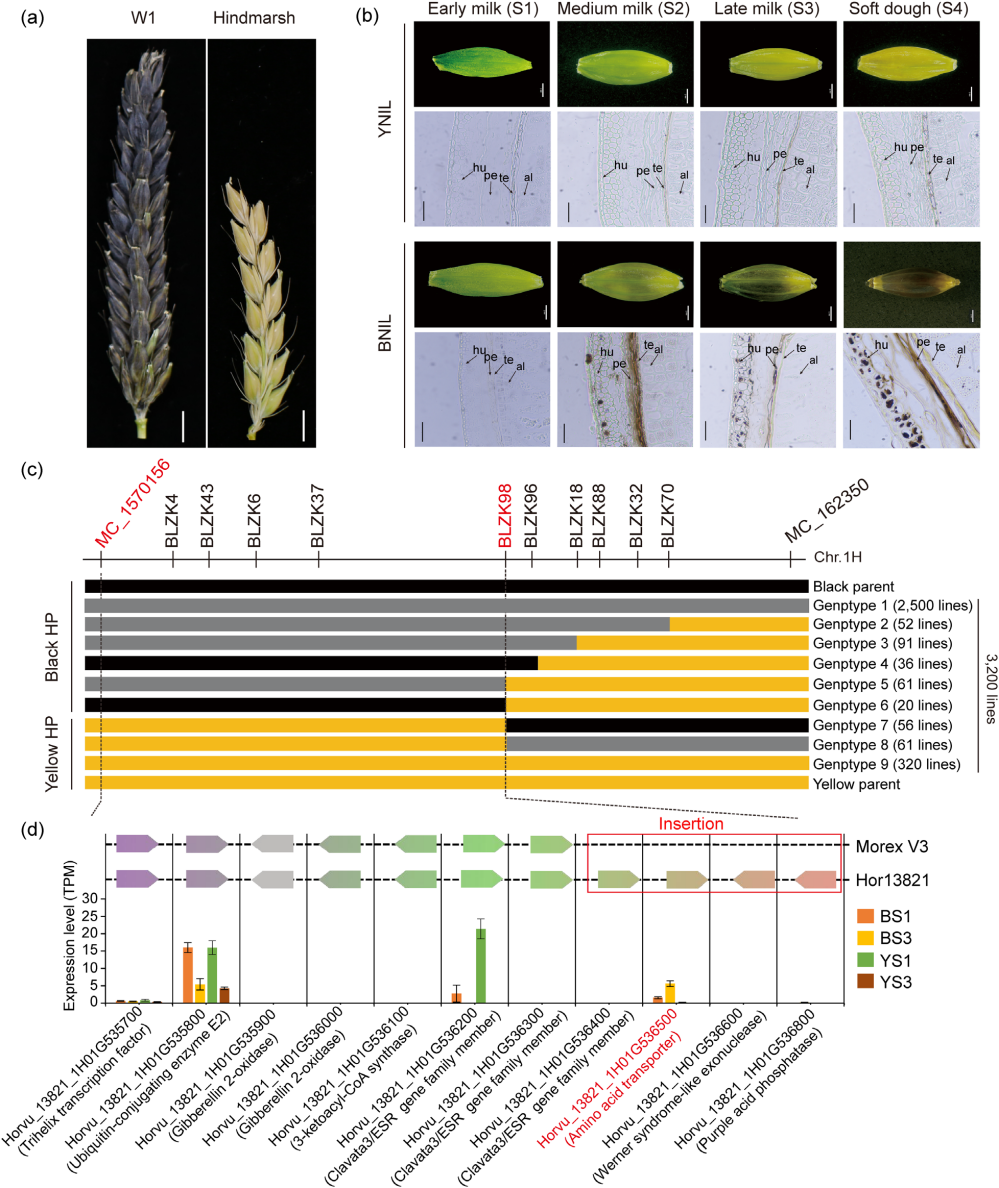
Map‐Based Cloning of *HvBlp*. (a) Spikes (awn removed) of W1 and Hindmarsh at soft dough stage; Scale bar denotes 1 cm; (b) Cross‐sections of grain set by YNIL and BNIL sampled at the early milk (S1), medium milk (S2), late milk (S3) and soft dough stages (S4). Scale bar for images of whole grains: 2 mm; and micrographs: 200 μm. Al: aleurone, te: testa, pe: pericarp and hu: husk; (c) Genetic linkage map of the *Blp* region based on two fine mapping populations. Black, yellow, and grey blocks represent the genotypes of homozygous W1, homozygous Hindmarsh and heterozygote, respectively. (d) Gene annotations and expression levels identified in the Hor13821 and Morex V3 genome references between the BLZK98 and MC_1570156 markers.

Two near‐isogenic lines (NILs) with distinct grain colours (black: BNIL; yellow: YNIL) were used to characterize the black husk/pericarp trait in barley. BNIL and YNIL are stable NILs derived from the segregation of a single BC_2_F_6_ plant (donor parent: a wild barley line W1 with black husk; recurrent parent: Australian barley cultivar Hindmarsh with yellow husk; Figure 1a). The black coloration was restricted to the husk/pericarp and awn tissues of W1 and BNIL. As shown in Figure 1b, both BNIL and YNIL displayed greenish husks at the early milk stage (S1) with no significant difference. At the medium milk stage (S2), a black hue emerged in the middle part of the BNIL husk while YNIL remained light greenish. At the late milk stage (S3), most parts of the BNIL husk turned light black, which became completely black at the soft dough stage (S4). In contrast, the husk of YNIL changed from light yellow at S3 to a complete yellow colour at S4. Histological analyses (Figure 1b) of horizontally sectioned seeds showed that black pigments started accumulating in the pericarps of BNIL at S2, which enhanced at S3 and S4. In comparison, no black pigment accumulation was observed in the seeds of YNIL throughout the seed development.

We screened 3200 inbred lines from our BC_2_F_6_ and TH1‐7 populations using previous markers (Long *et al*., 2019), plus 10 newly designed indel markers (Table S1), which enabled us to position *HvBlp* between markers BLZK98 and MC_1570156, spanning 0.484 Mb using Morex (V3) as reference (Figure 1d). Within this genetic interval, Morex (yellow husk) contains only seven annotated genes, none of which seem to be related to melanin or anthocyanin production. Thus, we searched the genome of a black husk genotype Hor13821 published in a recent pangenome study (Jayakodi *et al*., 2020). We found that Hor13821 contains 11 predicted genes within the mapped interval, spanning a 0.694 Mb region. In addition to the seven genes annotated in Morex, Hor13821 contains four additional genes, representing a genetic insertion. One of the four inserted genes *Horvu_13821_1H01G536500* encodes an amino acid transporter and shares 64.3% amino acid similarity with the previously characterized tyrosine transporter *OsBh4* in *Oryza. Rufipogon*, which was shown to be responsible for the black hull trait (Fukuda *et al*., 2012; Zhu *et al*., 2011). Protein structural analyses revealed a highly conserved integral amino acid transporter transmembrane domain PF01490 within *Horvu_13821_1H01G536500* and *OsBh4* (Figure S1). Thus, *Horvu_13821_1H01G536500* (referred to as *HvBlp* thereafter) was selected for further analyses.

**Figure 2:**
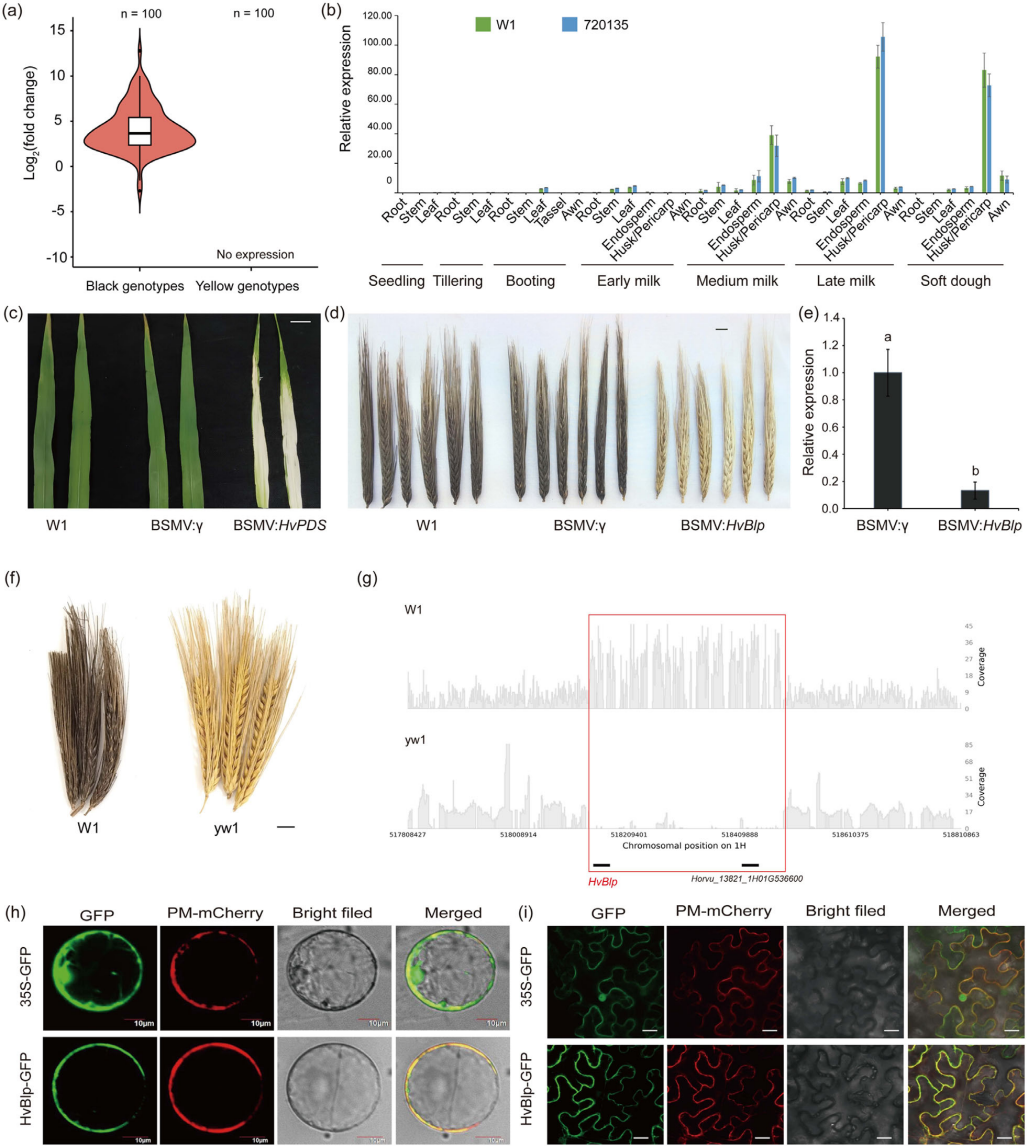
Validation of *Horvu_13821_1H01G536500* function. (a) Expression patterns of *Horvu_13821_1H01G536500* between 100 black husk/pericarp lines and 100 yellow husk/pericarp lines at the late milk stage. (b) Expression patterns of *Horvu_13821_1H01G536500* across 35 tissues at seven developmental stages of two black‐coloured barley (W1 and 720 135). (c) Barley leaf inoculated with BSMV:*HvPDS* shows white stripes, indicating efficient viral infection and gene silencing; scale bar, 1 cm. (d) A wild‐type spike inoculated with BSMV:*HvBlp* shows a discoloured phenotype; scale bar, 1 cm. (e) Silencing efficiency of *Horvu_13821_1H01G536500* in plants inoculated with BSMV:*HvBlp* and BSMV:γ (control); (a) and (b) above the bars indicated significant differences (*P* < 0.05) based on student's *t*‐test. (f) Displaying the phenotypes of mature spikes of W1 and yw1; scale bar, 1 cm. (g) Displays the read coverage in W1 and yw1 using Hor13821 as the reference; The potentially deleted region covering *HvBlp* was highlighted. Subcellular localization of *HvBlp*; Transient expression of the 35S‐GFP and HvBlp‐GFP fusion protein in barley leaf protoplasts (h) and *Nicotiana benthamiana* cells (i) with PM‐mCherry as a plasma membrane marker (pCAMBIA1300‐35S‐PM‐mCherry). Scale bars = 10 μm (h) and 20 μm (i). All error bars indicate standard deviation.

To validate *HvBlp*'s function in the black husk/pericarp trait, RNAseq was performed for the husk/pericarp tissues of YNIL and BNIL at the S1 and S3 stages. Out of the 11 candidate genes mapped in Hor13821, only *HvBlp* was highly and specifically expressed in the black‐coloured husk/pericarp tissues in BNIL at S3 stage: BS3, while the other 10 genes either were barely expressed or displayed no significant difference between BINL and YNIL (Figure 1d; Table S2). In addition, we analysed another published transcriptome datasets of different black‐ and yellow‐coloured barley lines (Table S2; Glagoleva *et al*., 2022) and confirmed that *HvBlp* was uniquely transcribed in the black barley genotype. Next, we performed qRT‐PCR on *HvBlp* in 100 black and 100 yellow inbred lines. Results showed that *HvBlp* was specifically expressed in the black husk/pericarp genotypes (Figure 2a). Further qRT‐PCR analyses in 35 tissues at seven developmental stages of two black‐coloured barley (W1 and 720135) revealed the spatial and temporal expression patterns for *HvBlp*, which was expressed the highest in the husk/pericarp tissues, moderately in awn, and slightly in leaf and endosperm at the medium milk (S2), late milk (S3) and soft dough (S4) stages (Figure 2b). Only weak expression was detected in the target tissues at the early milk stage (S1), consistent with the phenotype development. Based on these observations, we predicted that *HvBlp* may control the black husk/pericarp trait in barley.

### *HvBlp* silencing and deletion eliminated the black‐husk trait in barley

To validate *HvBlp*'s role in barley black colouration, we silenced *HvBlp* in the black‐coloured W1 using the BSMV–VIGS approach. Firstly, a reporter gene *HvPDS* was used to verify the BSMV–VIGS system. As shown in Figure 2c, the leaves of the BSMV:*HvPDS*‐inoculated plants displayed clear photobleaching symptoms 15 days post‐inoculation, whilst the leaves of BSMV:γ‐inoculated (mock) plants were not affected. For the mock (empty vector BSMV:γ) and BSMV:*HvBlp* experiments, the spike tissues, instead of leaves, were inoculated. As shown in Figure 2d, the spikes of the W1 wild‐type and its mock plants developed the normal black husk colour. In contrast, when *HvBlp* was tested, the spikes (Figure 2d right; 12 in total from different plants; six displayed, the other six used for RNA extraction) of W1 inoculated with BSMV:*HvBlp* displayed yellow or light grey husk colour. The knockdown of *HvBlp* was verified by qRT–PCR, which showed that the transcription of *HvBlp* in the BSMV:*HvBlp* inoculated spikes was reduced by 85.93% compared to that in the BSMV:γ‐inoculated (mock) spikes (Figure 2e). The BSMV–VIGS experiments were repeated twice using different batches of plant materials, consistent results were obtained (Figure S2), thereby supporting the involvement of *HvBlp* in the black husk trait.

To further confirm *HvBlp*'s function in the black husk trait, we identified a yellow husk/pericarp mutant line yw1 (Figure 2f) from a collection of 30 000 mutagenesis M_4_ lines created by gamma ray radiation of W1 and performed 17× whole‐genome long‐read sequencing (Table S3; Figure S3). A total of 3 904 186 clean reads were generated and mapped to the Hor13821 genome (Table S3). In addition, whole genome short‐gun sequencing data for the wild‐type W1 was downloaded from a previous study (Tan *et al*., 2020) and was mapped to the Hor13821 genome as well. Read coverage for a 1 Mb region covering the *HvBlp* locus was compared. As shown in Figure 2g, the wild‐type W1 displayed clearly increased read coverage for a genetic region spanning ~350 kb, covering two annotated genes: *HvBlp* and *Horvu_13821_1H01G536600*, implying a potential duplication of this fragment in W1 compared to that in Hor13821. In contrast, barely no read was detected for this region in the yw1 mutant line, suggesting a potential deletion induced by the gamma ray mutation, which may contribute to the yellow husk/pericarp trait. The deletion of *HvBlp* in yw1 was confirmed by PCR using multiple gene‐specific markers (data not shown). Taken together, these results provided direct evidence that *HvBlp* controlled the black husk/pericarp trait in barley.

To examine the subcellular localization of *HvBlp*, HvBlp‐GFP fusion protein was transiently expressed in barley protoplasts (Figure 2h) and *Nicotiana benthamiana* leaves (Figure 2i). Both experiments showed that the expressed *HvBlp* protein was targeted to the plasma membrane, consistent with its functional annotation as a membrane‐bound amino acid transporter and similar to that reported for *OsBh4* in rice (Zhu *et al*., 2011).

### *HvBlp* and *OsBh4* evolved from different phylogenetic lineages resulted from dispersed and tandem duplications, respectively

**Figure 3:**
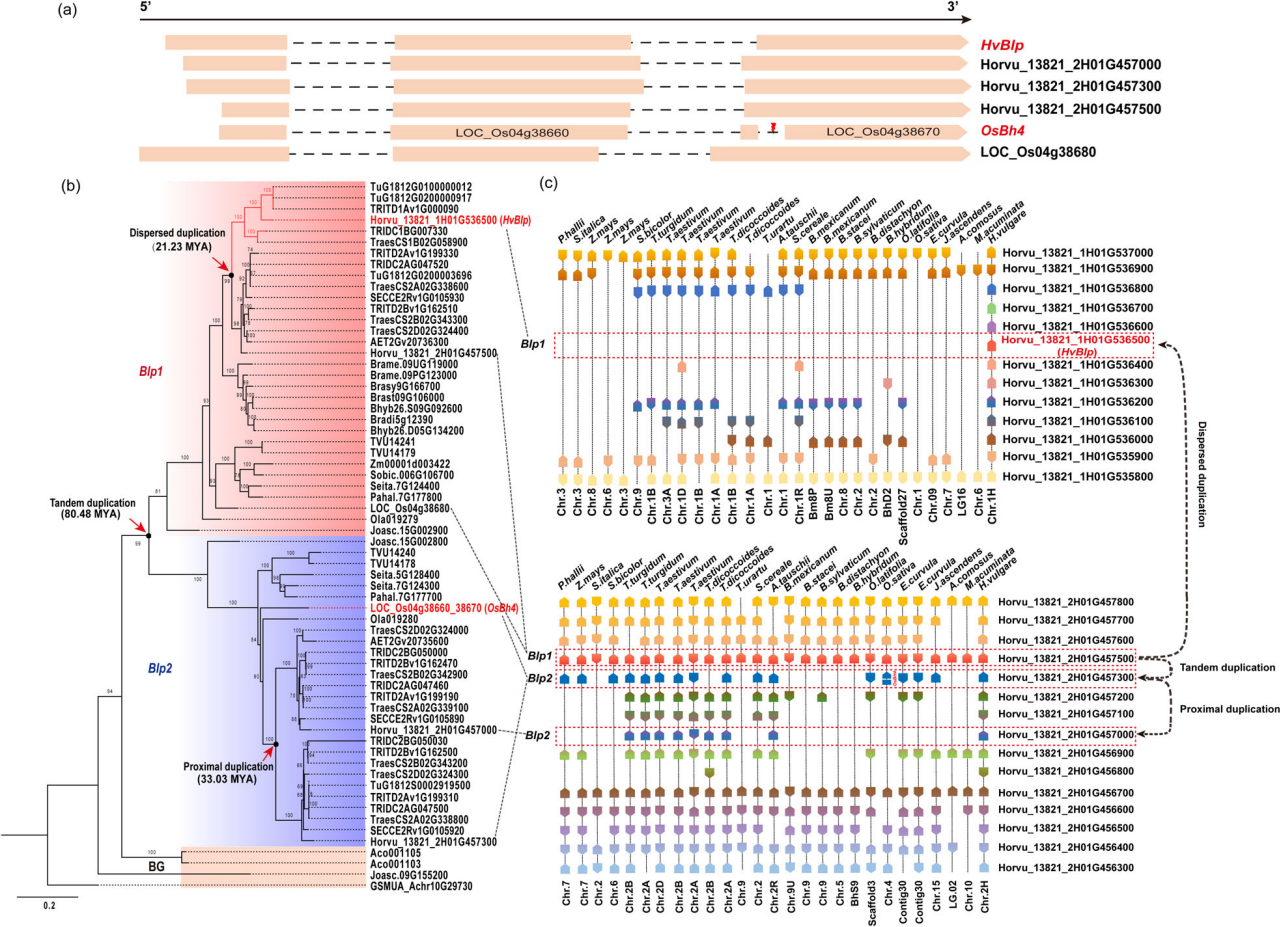
Phylogenetic profiling of *HvBlp* homologues. (a) Gene structure comparison of the *Blp1*‐, *Blp2*‐type genes in barley and rice. (b) Maximum likelihood phylogeny for the ATLb6 subgroup members in 22 monocot species; The *Blp1* and *Blp2* lineages were coloured in red and blue, respectively; four homologues from *Musa acuminata* (*GSMUA_Achr10G29730*), *Joinvillea ascendens* (*Joasc.09G155200*) and *Ananas comosus* (*Ac0001103* and *Ac0001105*) were included as background (BG). Bootstrapping support was labelled above each branch. The estimated divergence time was annotated for the corresponding nodes. (c) Displays the synteny of *Blp1* and *Blp2* homologues in 22 monocot species using barley Hor13821 genome as references.

To investigate the evolutionary origin of *HvBlp* in barley and its phylogenetic relationship with *OsBh4*, we performed a genome‐wide screening and phylogeny clustering of 4102 amino acid transporters (PF00324 and PF01490) in 42 plant genomes, covering Rhodophyta (3), Chlorophyta (1), Streptophyta (1), Bryophyte (2), Lycophytes (1), Angiosperms (2), Monocots (23) and Eudicots (9) (Figure S4a,b). Further, neighbour joining (NJ) phylogeny showed that *HvBlp* and *OsBh4* belonged to subgroup ATLb6, which contained 64 homologues from 22 monocot species (Figure S4c) and was thus identified as the direct *HvBlp* homologues. Within subgroup ATLb6, barley contains four genes: *Horvu_13821_1H01G536500* (*HvBlp*), *Horvu_13821_2H01G457000*, *Horvu_13821_2H01G457300*, *Horvu_13821_2H01G457500*), while rice has three annotated members: *LOC_0s04g38660*, *LOC_0s04g38670* and *LOC_0s04g38680*. Sequence alignment showed that *LOC_0s04g38660* and *LOC_0s04g38670* corresponded to the first and second halves of *OsBh4* (Figure 3a), which might be caused by an annotation error, due to a 22 bp deletion for *OsBh4* in the non‐black hull genotype (Zhu *et al*., 2011). Thus, we manually concatenated *LOC_0s04g38660* and *LOC_0s04g38670* as a single gene *Os04g38660_38670* in later phylogeny analyses.

A more robust maximum likelihood (ML) phylogeny based on the CDS sequences was constructed for the ATLb6 subgroup specifically (Data S1). In the ML phylogeny (Figure 3b), the single *HvBlp* homologue in *Musa acuminata* (*GSMUA_Achr10G29730*) diverged first, followed by one homologue from *Joinvillea ascendens* (*Joasc.09G155200*) and two from *Ananas comosus* (*Aco001103* and *Aco001105*), consistent with their species phylogeny. The rest *HvBlp* homologues evolved into two major clades (named *Blp1* and *Blp2*), implying an ancient duplication event in the common ancestor of these plants. Based on the species phylogeny, this duplication event corresponds to ~80.48 million years ago (MYA; Figure 3b). At least two *HvBlp* homologues were present in each of the 16 monocot genomes with the exception of *Musa acuminata* (*GSMUA_Achr10G29730*), *Brachypodium distachyon* (*Bradi5g12390*), *Brachypodium stacei* (*Brast09G106000*), *Brachypodium sylvaticum* (*Brasy9G166700*), *Sorghum bicolour* (*Sobic.006G106700*) and *Zea mays* (*Zm00001d003422*), which contains a single *HvBlp* homologue.

Interestingly, we noted that *HvBlp* and *OsBh4* belonged to lineages *Blp1* and *Blp2*, respectively, implying an independent origin. In addition to the *Blp2*‐type *OsBh4* (*Os04g38660_38670*), rice contains an additional *Blp1*‐type gene *Os04g38680*. Similarly, in addition to the *Blp1*‐type *Horvu_13821_1H01G536500 (HvBlp)*, barley contains another *Blp1*‐type *Horvu_13821_2H01G457500* and two *Blp2*‐type *Horvu_13821_2H01G457000* and *Horvu_13821_2H01G457300* (Figure 3b,c). Further synteny analyses showed that at least one pair of *Blp1* and *Blp2* genes in 14 (including *Joinvillea ascendens*, *Oriza sativa*, *Triticum aestivum* and *Hordeum vulgare*) out of the 22 target species were located in a tandem manner on their corresponding chromosomes (Figure 3c), providing direct evidence that *Blp1* and *Blp2* have resulted from a tandem gene duplication event. *Blp1* on 2H seems to be strictly preserved in a syntenic region in all target species, implying *Blp1* on 2H as the ancestor copy. The absence of the tandemly duplicated *Blp2* at the conserved syntenic region in some species like *Musa acuminata*, *Brachypodium hybridum* and *Zea mays* may be caused by gene loss, which is a common observation in gene evolution via duplication.

In contrast to rice, which contains a single copy of *Blp1* and *Blp2* each, *Triticeae* species including barley have experienced additional duplication events in both the *Blp1* and *Blp2* lineages (Figure 3b,c). Within the *Blp1* lineage, the subclade (highlighted in red) containing *HvBlp* (*Horvu_13821_1H01G536500*, *Blp1*‐type on 1H) represented a dispersed duplication (different chromosome and the lack of conserved synteny) from the ancient *Blp1* on 2H (Figure 3b). This subclade also included *TraesCS1B02G058900* (wheat), *TRIDC1BG007330* (emmer), *TRITD1Av1G000090* (durum wheat), *TuG1812G0100000012* and *TuG1812G020000917* (Urartu), which however are located on a different location not syntenic with *HvBlp* in barley (Figure 3c). The dispersed replication giving rise to *HvBlp* was dated at ~21.23 MYA (Figure 3b; Data S1), suggesting that the black husk/pericarp trait may have emerged in the common ancestor of *Triticeae*. In comparison to the *Blp1* lineage, the *Triticeae Blp2* lineage diverged further via a proximal duplication event, leading to two sister subclades (distanced by two inserted genes using barley genome as reference; Figure 3b,c).

### *HvBlp* and *OsBh4* displayed transcriptional convergence in the husk/pericarp tissues

**Figure 4:**
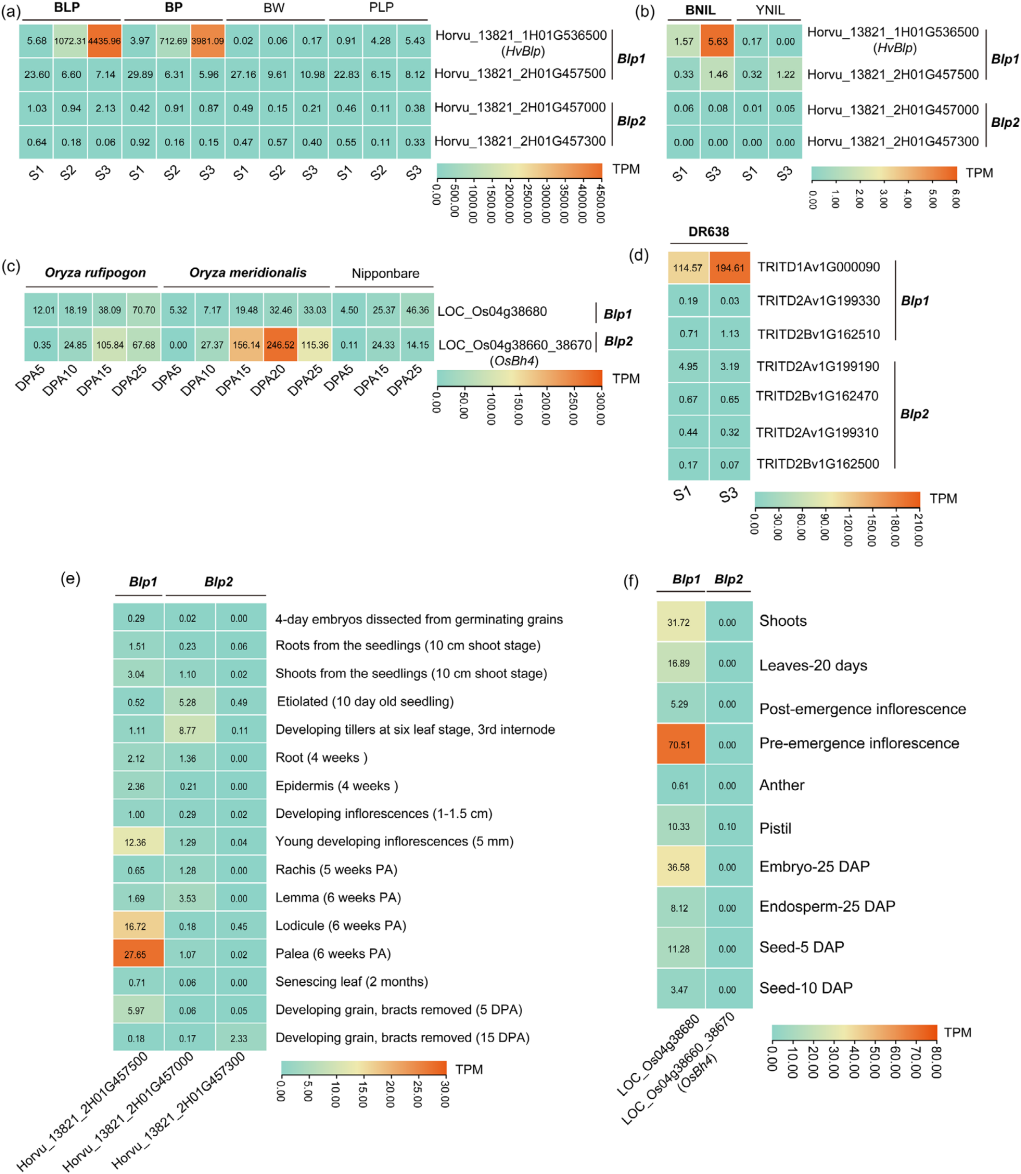
Expression patterns of *Blp1‐type* and *Blp2‐type* homologous genes. (a) The transcriptional expression of *Blp1* and *Blp2* homologous genes in the spikes of barley NILs BLP (black lemma and pericarp), PLP (purple lemma and pericarp), BP (black and purple) and of cv. Bowman (BW) at the booting stage (1), late milk stage (2), and early dough stage (3) (Glagoleva *et al*., 2022). (b) The transcriptional expression of *Blp1* and *Blp2* homologous genes in the husk/pericarp tissues of BNIL and YNIL at green (early milk stage, S1) and black grain stages (late milk, S3). (c) The transcriptional profiles of *Blp1* and *Blp2* homologous genes in the panicles of black‐coloured rice genotypes *Oryza meridionalis* and *Oryza rufipogon*, and non‐black genotype *Nipponbare* at several stages after days post anthesis (Hasan *et al*., 2022). (d) The expression levels of *Blp1* and *Blp2* homologous genes in the husk/pericarp samples of black‐coloured durum wheat at green (early milk stage, S1) and black grain stages (late milk, S3). (e) The expression levels of *Blp1* and *Blp2* homologous genes in 16 different organs or tissues of the non‐black genotype barley Morex from publicly available RNA‐seq database (http://202.194.139.32/). (f) The expression levels of *Blp1* and *Blp2* homologous genes in 10 different organs or tissues of non‐black genotype rice *Nipponbare* from publicly available RNA‐seq database (http://rice.uga.edu/index.shtml). The expression levels were estimated by transcripts per kilobase million (TPM).

*HvBlp* (*Blp1*‐type) and *OsBh4* (*Blp2*‐type) belong to different evolutionary lineages but share a common biological function in the black husk, implying a functional convergence at the gene transcription level. To verify this hypothesis, we re‐analysed public RNA seq data and characterized the transcriptional profiles of *Blp1*‐type and *Blp2*‐type genes in barley, durum wheat and rice with and/or without black colour. In barley (Figure 4a), *HvBlp* was actively expressed in black‐ and black‐purple‐coloured barley grains and strongly up‐regulated along with grain colour development, but not expressed in the yellow‐ and purple‐coloured genotypes. The other barley *Blp1* gene *Horvu_13821_2H01G457500* (putatively ancestral copy) displayed moderate to low expression in the grain tissues of all genotypes, whilst the two *Blp2* genes *Horvu_13821_2H01G457000* and *Horvu_13821_2H01G457300* were barely transcribed in grain tissues (Figure 4a). Similar expression patterns were observed in the black‐ and yellow‐coloured barley NILs lines, which showed *HvBlp* was only actively expressed in the grain tissues of the black‐husk genotype (Figure 4b). In rice (Figure 4c), the *Blp2*‐type *OsBh4* (*LOC_Os04g38660*_*38670*) was highly expressed in the grain tissues of the black‐coloured *Oryza meridionalis* and *Oryza rufipogon* but was barely transcribed in the non‐black genotype *Nipponbare*. In comparison, the rice *Blp1* homologue (*LOC_Os04g38680*) was only moderately or weakly expressed during grain development in all rice species, similar to that observed for the *Blp1*‐type *Horvu_13821_2H01G457500* in barley. Thus, we observed that *HvBlp* and *OsBh4* displayed similar expression in the grain tissues, supporting a scenario of transcriptional convergence. Further transcriptional data in black‐coloured durum wheat (Figure 4d) revealed a similar pattern for *TRITD1Av1G00090* (the direct *HvBlp* homologue), which was highly expressed and up‐regulated in the black‐coloured grain tissues, while the other *Blp* homologues were barely expressed. This suggests that *TRITD1Av1G00090* should be the black grain gene in durum wheat.

In addition to the grain tissues of black‐coloured genotypes, transcriptional data of barley and rice *Blp* homologues in various other tissues was also explored. In barley genome Morex where *HvBlp* is absent, the other *Blp1*‐type gene *Horvu_13821_2H01G457500* was actively transcribed in various tissues: the highest in palea, lodicule, and young inflorescences, while the *Blp2*‐type genes (*Horvu_13821_2H01G457000 and Horvu_13821_2H01G457300*) were weakly expressed in specific tissues only (Figure 4e). In rice reference genome where non‐functional (22 bp deletion) *OsBh4* is present, the *Blp1*‐type *LOC_Os04g38680* was also widely transcribed in various tissues (Figure 4f), similar to the *Blp1‐type* gene *Horvu_13821_2H01G457500* in barley (Figure 4e). In addition, rice *Blp2*‐type *OsBh4* (LOC_Os04g38660_38670) was barely expressed in any tissue (Figure 4f), similar to the barley *Blp2*‐type genes (Figure 4e).

Taken together, our results showed that, in non‐grain tissues, the transcription patterns of both *Blp1* and *Blp2* homologues were conserved across barley and rice, consistent with their phylogeny classification. However, in the black‐grain tissues, barley *Blp1*‐type *HvBlp* and rice *Blp2*‐type *OsBh4* displayed a similar and converged gene transcription pattern. These results indicated that the black husk trait in barley and rice has evolved independently as a convergent phenotype. The wide‐spread transcription of *Blp1* in various non‐grain tissues in both barley (on 2H) and rice (on Chr.4), together with a conserved synteny, supports that the *Blp1*‐type on 2H, not including *HvBlp*, is the potential ancestral copy.

### The *Blp1* phylogenetic lineage containing *HvBlp* has been affected by positive selection

To detect if there were any positive selection acting on *Blp1* and *Blp2*, the ratio (*ω*) of non‐synonymous (*Ka*) to synonymous (*Ks*) substitutions was calculated for the developed ML phylogeny, whereby *ω* < 1, *ω* = 1 and *ω* > 1 indicate purifying, neutral, and positive selections, respectively. Three phylogeny lineages: *Blp1*, *Blp2*, and background *Blp* (BG: highlighted in Figure 3b) were specified for *ω* calculation, corresponding to *ω*_*Blp1*_, *ω*_*Blp2*_ and *ω*_BG_, respectively. Under the Branch‐Specific method (Table 1), likelihood‐ratio tests (LRTs) showed that the three‐ratio model (*ω*_*Blp1*_ ≠ *ω*_*Blp2*_ ≠ *ω*_*BG*_) fitted the data better (ln *L* = −15671.12) than the one‐ratio model (*ω*_*Blp1*_ = *ω*_*Blp2*_ = *ω*_*BG*_, ln *L* = −15672.42) and two‐ratio (ln *L* = −15672.11 and − 15672.40 for *ω*_*Blp1*_ ≠ *ω*_*Blp2*_ = *ω*_*BG*_ and *ω*_*Blp2*_ ≠ *ω*_*Blp1*_ = *ω*_*BG*_, respectively) models. The *ω* values of *ω*_*Blp1*_, *ω*_*Blp2*_ and *ω*_*BG*_ in the three‐ratio model were 0.09987, 0.29023 and 0.12180, respectively, indicating that *Blp1* lineages were under strong purifying selection, while the *Blp2* lineage is relatively more relaxed (Table 1). This observation is consistent with the indication that *Blp1* is the ancestral copy under stronger functional constraints. To test if positive selection may have acted on specific amino acid sites, we applied the Branch‐Site models, which allow *ω* to vary across both branches and amino acid sites. Results showed that 11 amino acid sites (9G, 67S, 105V, 127M, 130F, 149I, 155N, 203E, 210L, 302S and 313I; numbered according to *HvBlp*) in the *Blp1* lineage were found to be under positive selection (*ω*_2_ = 18.04453; Table 1). Comparison with the neutral site‐specific model M1 showed that these sites are indeed under positive selection in the *Blp1* lineage. In contrast, no positive selection site was detected when the *Blp2* lineage was tested (Data S2).

**Table 1:** Natural selection tests on *Blp* genes.

Model	np	*l* = ln *L*	Estimates of parameters	Positively selected sites
One‐ratio				
*ω*_*Blp1*_ = *ω*_*Blp2*_ = *ω*_*BG*_	1	−15672.42	*ω*_*Blp1*_ = *ω*_*BLP2*_ = *ω*_*BG*_ = 0.12218	Not allowed (NA)
Branch‐specific models				
*ω*_*Blp1*_ ≠ *ω*_*Blp2*_ = *ω*_*BG*_ (2 ratio)	2	−15672.11	*ω*_*Blp1*_ = 0.09912 *ω*_*Blp2*_ = *ω*_*BG*_ = 0.12298	NA
*ω*_*Blp2*_ ≠ *ω*_*Blp1*_ = *ω*_*BG*_ (2 ratio)	2	−15671.40	*ω*_*Blp2*_ = 0.29108 *ω*_*Blp1*_ = *ω*_*BG*_ = 0.12105	NA
*ω*_*Blp1*_ ≠ *ω*_*Blp2*_ ≠ *ω*_*BG*_ (3 ratios)	3	−15671.12	*ω*_*Blp1*_ = 0.09987 *ω*_*Blp2*_ = 0.29023 *ω*_*BG*_ = 0.12180	NA
Branch‐site‐specific models (selection test of *BLP1* as the foreground lineage)				
Model A (4 site classes)	4	−15444.71	*p*_0_ = 82.244%, *p*_1_ = 11.503% (*p*_2_ + *p*_3_ = 6.253%); *ω*_0_ = 0.09624, (*ω*_1_ = 1.0), *ω*_2_ = 18.04453	Positively selected sites in *Blp2*: 9G 0.943, 67S 0.824, 105V 0.553, 127M 0.786, 130F 0.648, 149I 0.905, 155N 0.764, 203E 0.881, 210L 0.888, 302S 0.723, 313I 0.978
Model A Null (4 site classes)	3	−15446.57	*p*_0_ = 69.551%, *p*_1_ = 9.739% (*p*_2_ + *p*_3_ = 20.71%), *ω*_0_ = 0.09635 (*ω*_1_ = 1.0, *ω*_2_ = 1.0)	NA

In one‐ratio and Branch‐specific models, *ω*_Blp1_, *ω*_Blp2_ and *ω*_BG_ stand for *Ka/Ks* values for *Poaceae Blp1*, *Poaceae Blp2* and *non‐Poaceae Blp* branches in Figure 3. In the Site‐specific model M1, two site classes were specified: highly conserved sites (*ω*_0_) and neutral sites (*ω*_1_ = 1). For the Branch‐site models, *Blp1* was specified as the foreground group. In the Branch‐site model A, four site classes were specified. The first two classes have *ω* ratios of *ω*_0_ and *ω*_1_ respectively, corresponding to highly conserved sites and neutral sites across all lineages. In the other two site classes, the background lineages have *ω*_0_ or *ω*_1_ while the foreground lineages have *ω*_2_. p_0_, p_1_ and p_2_ represent the percentages of the corresponding site classes. np: number of parameters. L: likelihood value. Amino acid sites were numbered according to *HvBlp* (*Horvu_13821_1H01G536500*) in barley.

### Metabolome and transcriptome analyses in black‐coloured barley grains

**Figure 5:**
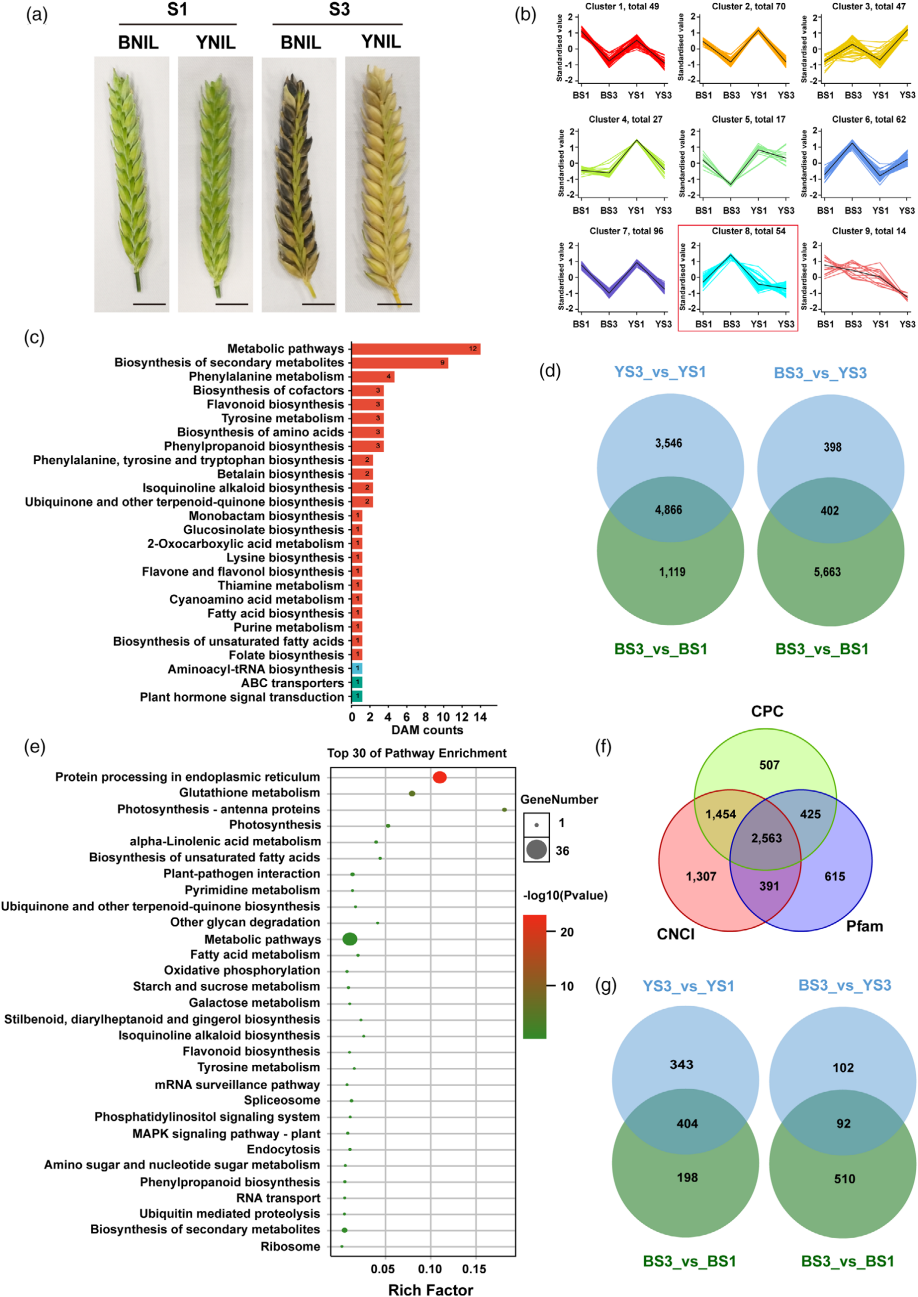
Analysis of Differentially accumulated metabolites (DAMs) as a function of the black husk/pericarp phenotype in barley seeds. (a) Representative images from BNIL (black husk/pericarp phenotype) and YNIL (yellow husk/pericarp genotype) spikes (awn removed) at two developmental stages for metabolome analysis; Scale bar, 1 cm. (b) *K*‐means clustering of all DAMs; Abscissae represent the sample and the ordinates represent the relative metabolite content (*Z*‐score normalized). (c) KEGG analysis of 54 DAMs in cluster 8. (d) The Venn diagram of DETs in comparisons of BS3_vs_BS1 and YS3_vs_YS1 and BS3_vs_BS1 and BS3_vs_YS3. (e) KEGG analysis of 402 DETs from the comparison of BS3_vs_YS3 and BS3_vs_BS1. (f) LncRNA identification using CPC, CNCI and Pfam databases. (g) The Venn diagram of DELs in comparisons of BS3_vs_BS1 and YS3_vs_YS1, and BS3_vs_BS1 and BS3_vs_YS3.

To gain insights into the biochemical mechanisms of the black husk/pericarp trait in barley, we performed both metabolome and transcriptome analyses on the husk/pericarp tissues of BNIL and YNIL at 2 developmental stages S1 (samples: BS1/YS1) and S3 (samples: BS3/YS3; Figure 5a). At the metabolome level, a total of 1072 metabolites were detected (Table S4). Comparative metabolite analyses identified 300, 113, 28 and 287 differentially accumulated metabolites (DAMs) for BS3_vs_BS1, BS3_vs_YS3, BS1_vs_YS1 and YS3_vs_YS1, respectively. Nine accumulation patterns of DAMs across the target samples were identified (Figure 5b), of which, cluster 8 contains 54 DAMs that were specifically up‐regulated in BS3, thus representing target metabolites related to black colouration. These target DAMs included 15 phenolic acids, 13 flavonoids, seven amino acids and derivatives, five alkaloids, and four organic acids (Table S5). KEGG enrichment analysis showed that these 54 DAMs were enriched in 26 pathways, including ‘metabolic pathways’, ‘Biosynthesis of secondary metabolites’, ‘flavonoid biosynthesis’, ‘tyrosine metabolism’ and ‘phenylpropanoid biosynthesis’ (Figure 5c). The enrichment and upregulation of ‘tyrosine metabolism’ related metabolites, such as phenolic acids, amino acids and their derivatives, in BS3, is in agreement with the predicted function of *HvBlp* as a putative tyrosine amino acid transporter.

At the transcriptome level, a total of 47 477 transcripts were identified. Differentially expressed transcripts (DETs) analyses revealed 4866 DETs commonly regulated for BS3_vs_BS1 and YS3_vs_YS1, which may be associated with the normal husk/pericarp development in barley (Figure 5d). Notably, 402 common DETs (Figure 5d), including *HvBlp*, were identified in both BS3_vs_YS3 and BS3_vs_BS1, which might be associated with melanin accumulation in barley husk/pericarp tissue. KEGG analysis showed that these 402 DETs are mainly involved in ‘protein processing in endoplasmic reticulum’, ‘glutathione metabolism’, ‘metabolic pathways’, ‘tyrosine metabolism’, ‘endocytosis’, ‘flavonoid biosynthesis’ and ‘phenylpropanoid biosynthesis’ pathways (Figure 5e), which generally overlapped with that detected in the metabolome enrichment analyses. Our metabolome and transcriptome data further highlighted ‘tyrosine metabolism’ as the critical biological pathway underlying the black husk/pericarp trait formation in barley.

In addition to protein‐coding RNA, long non‐coding RNA (lncRNA) has also been known to play an important role in genetic regulation. Using the Hor13821 genome as a reference, a total of 2563 lncRNAs were identified (Figure 5f). Among these, 209 and 195 were found as differentially expressed lncRNAs (DELs) between BNIL and YNIL at the S1 and S3 stages, respectively (Figure 5g). Particularly, 93 were detected at both S1 and S3 (Figure 5g) and were selected as candidate DELs that may affect the black husk/pericarp phenotype.

### Identifying the key factors involved in barley melanin biosynthesis via co‐expression analysis

**Figure 6:**
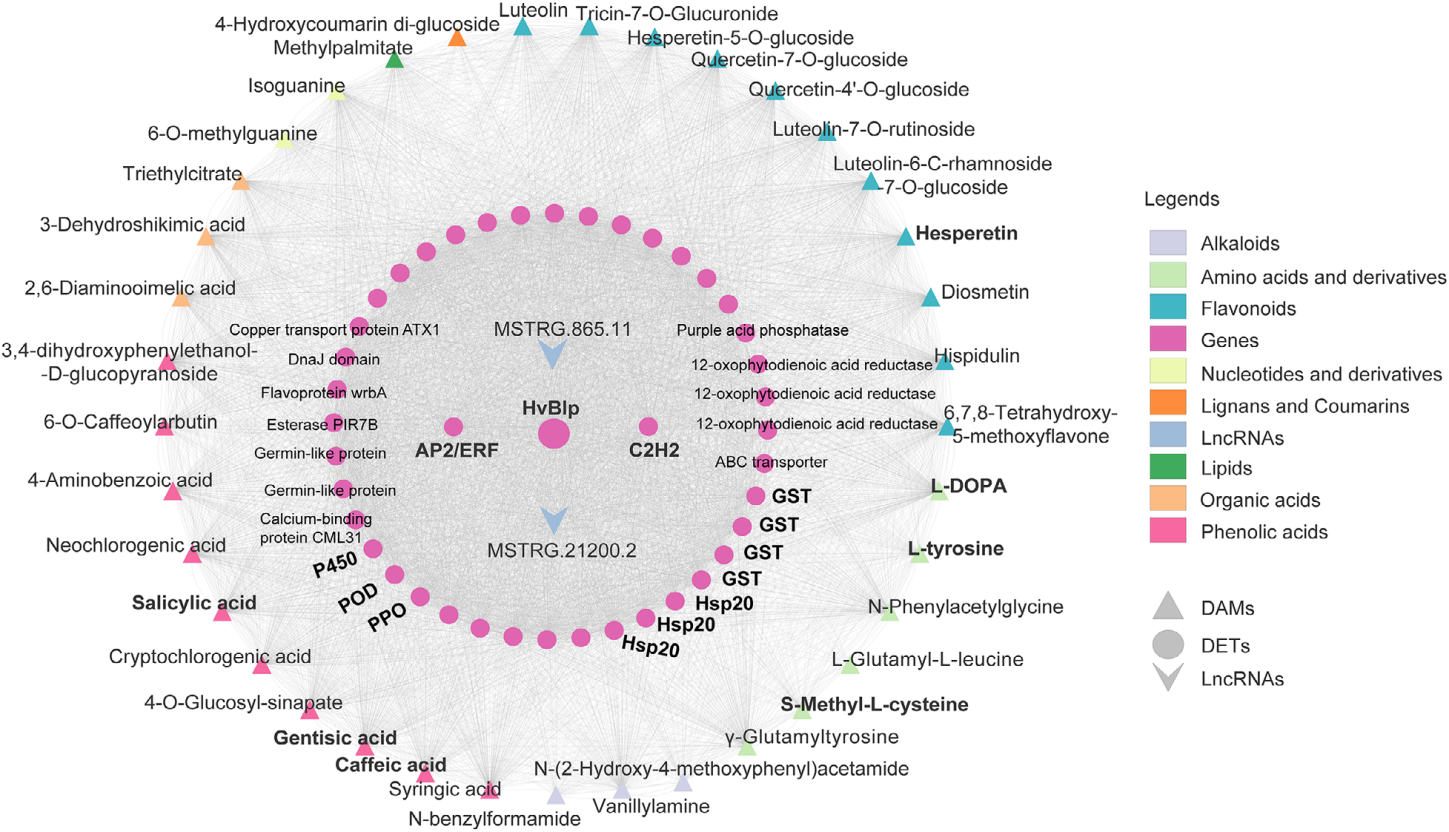
lncRNA–mRNA‐metabolite co‐expression network of barley black husk/pericarp trait.

Multi‐omics co‐expression network is a powerful tool to unravel complex regulatory pathways. Based on the metabolome and transcriptome data, we built a lncRNA–mRNA‐metabolite co‐expression network for the above‐identified 54 DAMs, 402 core DETs and 93 *Blp_lncRNAs*. Results showed that 37 DAMs, 42 DETs and 2 *Blp_lncRNAs* were significantly and positively associated with the expression of *HvBlp* (PCC > 0.9, *P* < 0.01; Table S7), suggesting that they were tightly relevant with barley melanin formation (Figure 6). Specifically, these 37 DAMs, such as 3‐Dehydroshikimic acid, L‐tyrosine, L‐DOPA, S‐Methyl‐L‐cysteine, Gentisic acid, Caffeic acid, Salicylic acid and Hesperetin, fell into eight categories: flavonoids (11), phenolic acids (10) and amino acids and derivatives (6), alkaloids (3), organic acids (3), nucleotides and derivatives (2), lipids (1) and lignans and coumarins (1) (Table S6). Of these, the content of L‐tyrosine was significantly correlated with the expression level of *HvBlp*, implying that *HvBlp* functions as an L‐tyrosine transporter, which is similar to the observation of *OsBh4* (Zhu *et al*., 2011). Among the identified 42 DETs, 3 candidate genes encoding polyphenol oxidase (PPO) (*novel2290.t1*), cytochrome P450 (*Horvu_13821_1H01G537900.1*) and peroxidase (POD) (*Horvu_13821_3H01G432900*), respectively (Figure 6), were identified for their oxidative functions, highly consistent with previous suggestions that PPO is critical for melanin production (Glagoleva *et al*., 2020; Hasan *et al*., 2022). In addition, three heat shock protein 20 (HSP20) (*Horvu_13821_3H01G128600*, *Horvu_13821_3H01G128700* and *Horvu_13821_4H01G094500*) and four glutathione S‐transferases (GSTs) (*Horvu_13821_1H01G293500*, *Horvu_13821_3H01G577000*, *Horvu_13821_4H01G304400* and *Horvu_13821_5H01G571200*), involving in ‘protein processing in endoplasmic reticulum’ and ‘glutathione metabolism’ pathways, respectively, were identified, which may be involved in metabolite translocation. Furthermore, two DETs encoding AP2/ERF (*Horvu_13821_1H01G379000*) and C2H2 (*Horvu_13821_5H01G633600*) transcription factors and two DETs encoding lncRNAs (MSTRG.21200.2 and MSTRG.865.11) displayed strong correlations with *HvBlp*, suggesting that they might be involved in the regulation of *HvBlp* transcriptional expression (Figure 6).

## Discussion

### The black husk/pericarp trait in barley is caused by a gene insertion variation absent in the reference genome

The candidate gene controlling the black husk/pericarp trait in barley has puzzled barley researchers for a long time. Several previous studies (Bungartz *et al*., 2016; Glagoleva *et al*., 2017, 2022; Jia *et al*., 2017; Liu *et al*., 2023; Long *et al*., 2019; Shoeva *et al*., 2016) have attempted to uncover its molecular basis but have all failed to date. In the present study, we identified the corresponding candidate gene *HvBlp* by screening an exceptionally large collection of 3200 inbred lines. *HvBlp* encodes a putative membrane‐bound tyrosine transporter homologous to *OsBh4* controlling the same trait in rice. We confirmed *HvBlp*'s function by gene silencing, whole‐genome resequencing of a yellow hush/pericarp mutant yw1, and gene transcriptional analyses. We found that the black husk/pericarp trait in barley was caused by a gene‐insertion variation absent in the reference genome Morex. Our study was facilitated by the recent publication of 20 barley pangenomes, which includes the genome assembly of a black‐coloured barley line Hor13821 (Jayakodi *et al*., 2020). This highlights the limitation of using a single reference genome for genetic studies of important agronomic traits. Interestingly, resequencing data analyses showed that the genetic fragment containing *HvBlp* seems to have been duplicated in W1 compared to Hor13821. It is worthy for future studies to investigate if barley lines with more than one copy of *HvBlp* may have enhanced black colouration than those lines with a single copy, i.e. a gene dosage effect. Due to its easy‐characterized appearance and simple genetic basis (controlled by a single dominant locus), the black husk/trait has been widely used as a biological marker in barley breeding (Costa *et al*., 2001) and also as a model to validate various genetic techniques (Bungartz *et al*., 2016; Jiang *et al*., 2022). Thus, the identification of *HvBlp* not only provided an answer to a long‐sought biological question but also had important practical implications for future barley breeding and genetic studies. In addition to *HvBlp*, we also identified its orthologous genes in different wheat genomes. Particularly, *TRITD1Av1G000090* was shown to be highly expressed in black‐coloured durum wheat and needs further functional verification.

### Transcriptional convergence of *HvBlp* and *OsBh4* leads to the independent evolution of the black husk/pericarp trait in barley and rice

Given the relatively close relationship between barley and rice, both in the *Poaceae* family, one would assume the black husk/pericarp trait in these two plants should have the same genetic origin. In this study, we found that *HvBlp* is highly homologous to *OsBh4* (Zhu *et al*., 2011), implying a similar genetic and metabolic basis. However, synteny and phylogenetic analyses in this study showed clearly that *OsBh4* and *HvBlp* belong to different gene lineages, resulted from a tandem duplication (dated at 80.48 MYA in the common ancestor of *Poaceae*) and a dispersed duplication (dated at 21.23 MYA in the common ancestor of *Triticeae*), respectively, supporting an interesting case of convergent evolution. Synteny, phylogeny, and transcriptional analyses all indicated that the *Blp1* lineage genes located in the conserved syntenic region on 2H should be the ancestral gene copy, with *OsBh4* and *HvBlp* being sequentially duplicated, followed by convergent transcription in the husk/pericarp tissues. Despite the potential prevalence of phenotypic convergence in plants (Trewavas, 2014), only a limited number of well‐characterized cases have been reported to date. Compared to divergent evolution, convergent phenotypes and their underpinning molecular basis are generally more difficult to identify, partly because of the need to verify their independent origins. Reconstruction of the phenotype along the phylogeny has been suggested as an effective approach to identify convergent evolution (Arbuckle *et al*., 2014). In our analyses, we included five *Brachypodium* species intentionally to examine the putative ancestral status of the *Blp1* lineage. No black husk/pericarp trait or corresponding candidate gene seems to have evolved in these species. We further confirmed our conclusion by searching the published 54 *Brachypodium* pangenomes (Gordon *et al*., 2017) and also by personal communication with the authors who have not noticed any black husk trait in their collection of over 100 diverse germplasm lines.

Most phenotypic convergence have been related to environmental adaptation, such as the repeated emergence of C4 photosynthesis for adaptation to drought and high temperature (Heyduk *et al*., 2019), perenniality for seasonal environments (Hu *et al*., 2003), flower scents for specific pollinator (Knudsen and Tollsten, 1995), lateral shoot organ for terrestrial environments (Naramoto *et al*., 2019) and root organic acid secretion for aluminium tolerance (Ryan and Delhaize, 2010). In this study, we argue that the repeated emergence of the black husk/pericarp traits in barley and rice may also have a natural selection cause, potentially driven by similar abiotic stressors such as drought, high temperatures and strong lights. Indeed, we detected positive natural selection in the *Blp1* lineage. The failure to detect positive selection in the *Blp2* lineage (containing *OsBh4*) may indicate that the selection on the black husk/trait may mainly occur in the gene expression level, possibly in the gene promoter region. It is well‐known that plant melanins can protect seeds from insects, pathogen and mechanical damage (Choo *et al*., 2015; Glagoleva *et al*., 2020; Jocković *et al*., 2020). In addition, black‐coloured barley and rice generally have the highest antioxidant capacity compared to other coloured, due to enriched accumulation of melanins, phenolics and flavonoids (Ge *et al*., 2021; Glagoleva *et al*., 2022; Shen *et al*., 2016). In addition to natural selection, genetic and metabolic constraints have also been noted as another major contributing factor to phenotypic convergence (Christin *et al*., 2010). The black husk/pericarp trait in barley and rice share similar genetic and metabolic basis, which may result from the limited evolutionary paths for melanin production in plants. In this study, convergent transcription seems to be the direct cause of the black husk/pericarp trait convergence. Gene expression convergence has been associated with several recent cases of phenotypic convergence in plants (Cossard *et al*., 2022; Xu *et al*., 2020; Zhang *et al*., 2022). However, what distinguished the convergent evolution of the black husk/trait is that it involves repeatedly duplicated genes from different lineages. The underlying cause of this transcriptional convergence may be the genetic redundancies after gene duplication. This implication is consistent with a recent study in vertebrates (Foster *et al*., 2022) which suggest that redundancies in gene function may enable the repeated evolution of similar trait, i.e. convergent evolution. Since functional redundancy resulting from the duplication of similar genes (as in our case) is prevalent in plant genomes, we expect more similar cases of convergent evolution as reported in this study will be uncovered in the future.

### Proposed metabolic pathway and candidate genes for melanin biosynthesis in barley

In addition to its evolutionary significance, the identification of *HvBlp* in this study also marked a significant advance in our understanding of the genetic and metabolic pathways of melanin biosynthesis in plants. In addition to *HvBlp* and *OsBh4*, a homologous gene to *OsBh4* has also been shown to be upregulated in black‐coloured oats (Liu *et al*., 2021). It would be interesting for future studies to examine the genetic basis and evolutionary origin of the black husk/pericarp trait in this species. The putative function of *HvBlp* and *OsBh4* as potential tyrosine transporters is consistent with a recent transcriptome and metabolome study (Glagoleva *et al*., 2022), which highlighted the involvement of phenylpropanoid biosynthesis pathway in melanin production in black‐coloured barley. Similar reports have been made for melanin synthesis in *Zanthoxylum bungeanum* (Fei *et al*., 2021) and oat (Liu *et al*., 2021). Indeed, tyrosine has been shown as the precursor of all phenylpropanoids biosynthesis, which branched into different pathways for the production of flavonoids, melanins and other types of phenolic acids (Barros and Dixon, 2020; Cao *et al*., 2021; Fei *et al*., 2021; Singh *et al*., 2021). Given the highly conserved amino acid transporter transmembrane domain PF01490 in both *HvBlp* and *OsBh4* and their close relationship, it is highly likely that *HvBlp* may function as a tyrosine transporter in barley, which is consistent with the co‐expression analysis result (Figure 6). Future studies are needed to validate the in‐vitro tyrosine transporting activity for *HvBlp*. Consistently, we detected ‘flavonoid biosynthesis’, ‘tyrosine metabolism’ and ‘phenylpropanoid biosynthesis’ as differentially expressed pathways in our comparative transcriptome and metabolome analyses. We identified candidate genes and metabolites specifically associated with the transcription of *HvBlp*, which further confirmed the critical role of phenylpropanoids pathway in melanin production. The DAMs identified in this study using the ultra‐high performance liquid chromatography (UPLC)–MS/MS method were generally reliable and are consistent with other studies, but may need to be validated using an alternative method to confirm their potential involvement in melanin biosynthesis.

**Figure 7:**
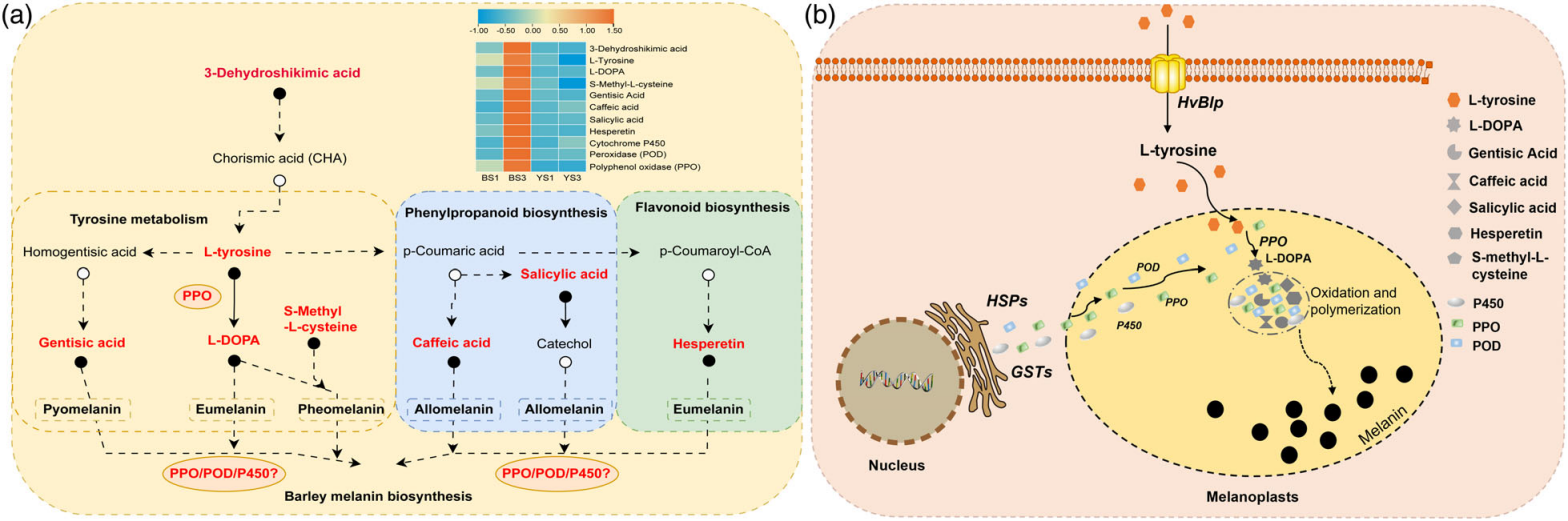
A tentative schematic model (a) and schematic representation (b) of the melanin biosynthesis in barley.

Based on metabolome results from this study, we highlighted the potential involvement of eight phenolic acids (3‐Dehydroshikimic acid, L‐tyrosine, L‐DOPA, S‐Methyl‐L‐cysteine, Gentisic acid, Caffeic acid, Salicylic acid and Hesperetin), three candidate genes (PPO, P450, POD) with oxidative functions, three HSP 20 genes with protein processing function and four GSTs with substance transport function, which enabled us to propose a detailed regulatory pathway for melanin production in barley (Figure 7a). Putatively, 3‐Dehydroshikimic acid serves as the precursor of L‐tyrosine production using Chorismic acid as the intermediate (Figure 7a). The synthesized L‐tyrosine is then transported into the cells of the husk/pericarp tissues by *HvBlp* embedded in the plasma membrane. Once inside the cell, the enriched L‐tyrosine passes through the permeable membrane of the chloroplasts where they are further converted into L‐DOPA by PPO activity for melanin production (Figure 7b). In addition, the enriched S‐Methyl‐L‐cysteine and L‐DOPA can also lead to pheomelanin production. Within the phenylpropanoid metabolic pathway, Caffeic acid and Salicylic acid were significantly up‐regulated in the black samples, which are precursors of allomelanin production, putatively catalysed by P450 activity. Lastly, within the flavonoid pathway, the enriched Hesperetin could be used for eumelanin production. In addition to PPO, P450 and POD, other enriched DETs including HSPs and GSTs have been reported to be involved in melanin biosynthesis as well (Figure 7b; He *et al*., 2021; Ibarrola‐Villava *et al*., 2012).

Depending on the chemical composition and structural features, melanins have been classified into three types: eumelanin, pheomelanin, and allomelanin (Glagoleva *et al*., 2020). Previously, it was thought that plant melanins belong to allomelanin which is devoid of nitrogen (Glagoleva *et al*., 2020), despite a recent report that all three types (allomelanin, eumelanin and pheomelanin) are present in a medicinal plant *Echinacea purpurea* (Kurkiewicz et al., 2022) and nitrogen‐containing melanins may also be present in barley (Shoeva *et al*., 2020). Our proposed pathway of melanin formation in barley seem to support the presence of multiple types of melanin as well, which may worth further attention because different types of melanin have been shown to retain varied biochemical properties (Guo *et al*., 2023).

In addition to *HvBlp*, we also identified three candidate genes significantly associated with *HvBlp* transcription. Of these, the gene encoding a putative PPO is of particular interest and needs further verification in future studies. In fact, PPO has been suggested to be responsible for melanin production in rice (Fukuda *et al*., 2012), sesame (Wei *et al*., 2016), bitter gourd (Zhong *et al*., 2022), persimmon (Qi *et al*., 2020) and watermelon (Li *et al*., 2019). The significance of lncRNA in diverse biological processes is crucial in plant kingdoms, which can modulate the expression of target genes through *cis*‐ and *trans*‐acting mechanisms; impact the structure and function of chromatin; and participate in RNA transcription, splicing and stabilization (Zheng *et al*., 2023). Several studies have illustrated the significance of lncRNAs in plant development and colour formation process (Li *et al*., 2023; Zheng *et al*., 2023). In this study, two Blp_lncRNAs were identified to be significantly co‐expressed with *HvBlp*, which may be involved in the stage‐ and tissue‐specific expression of *HvBlp*. Despite these new insights, our understanding of melanin synthesis in barley at the tissue and subcellular levels still needs further examination. Particularly, in addition to the well‐supported functional annotations, future studies are needed to validate the function of those candidate genes identified based on DETs, DEGs and DELs. In addition, a recent study reported the existence of a barley line ‘Hatiexi’ whose entire body including leaves, stems, awn, lemma and aleurone could turn black when the plant matures (Jiang *et al*., 2022). It would be interesting to examine the potential involvement of *HvBlp* in this barley line. In addition, Shoeva et al. showed that melanin formation in barley occurred in chloroplast‐derived plastids (Shoeva *et al*., 2020). Similar report in *Piptocarpha axillaris* also supported the role of plastid in phytomelanin synthesis (Coutinho *et al*., 2021). However, in contrast to barley, melanins in *Asparagales* and *Asteraceae* plants have been known to be secreted to the extra‐cellular space where they forms a distinct phytomelanin layer (Coutinho *et al*., 2021). The unique accumulation of melanin synthesis in the chloroplast in barley seems to be consistent with the subcellular location of PPO which has been suggested as a chloroplast protein (Glagoleva *et al*., 2020). However, we found that *HvBlp* was targeted to the plasma membrane, similar to the observation with *OsBh4* (Zhu *et al*., 2011). Further studies, such as the transport activity of *HvBlp* via yeast transformation and complementation, are needed to understand how the imported tyrosine passes the plastid membrane.

## Experimental procedures

### Histological analysis of barley seeds during the grain filling by paraffin sections

Two stable NILs: BNIL (black husk/pericarp) and YNIL (yellow husk/pericarp) derived from a single BC_2_F_6_ plant, with parental lines as W1 (black husk/pericarp, donor parent) and Hindmarsh (yellow husk/pericarp, recurrent parent), were grown under greenhouse conditions with a 14 h/10 h and 25 °C/21 °C day/night light and temperature cycle. More than 10 seeds of each biological replicate were randomly collected at 10 (early milk stage S1), 15 (medium milk stage S2), 20 (late milk stage S3) and 35 (soft dough stage S4) days past flowering (DAF). Seed samples were fixed in 3.7% (v/v) Formalin‐Acetic Acid‐Alcohol solution (FAA), then subjected to a series of dehydration and infiltration and embedded in paraffin as described by Cheng *et al*. (2023). The tissues were sectioned into 10‐μm sections using a microtome and observed under the microscope after deparaffinization.

### Map‐based cloning of *HvBlp*

Inbred lines (300 BC_2_F_3_, 400 BC_2_F_4_, 1000 BC_2_F_5_ and 500 BC_2_F_6_) from Hindmarsh and W1, together with 1000 residual heterozygous lines from a segregating population (TH1‐7) were used for genetic mapping. Insertion/deletion (Indel) markers were designed using an in‐house barley server (http://146.118.64.11/BarleyVar/; Tan *et al*., 2020). Grain colour was assessed visually. Candidate genes in the fine‐mapped interval were analysed for Morex V3 (yellow husk/pericarp; Mascher *et al*., 2021) and Hor13821 (black husk/pericarp; Jayakodi *et al*., 2020).

### Quantitative real‐time PCR (qRT–PCR) analysis

The husk/pericarp tissues from BC_2_F_6_ and TH1‐7 populations were isolated at stage S3. In addition, roots, stems, leaves and awns tissues of black husk/pericarp barley genotypes (W1 and 720 135) were collected at seedling, tillering, booting, early medium milk, late milk and soft dough stages. RNA was extracted using TRIzol reagent (Tiangen, Beijing, China), and cDNA was synthesized using HiScript III RT SuperMix (Vazyme Biotech, Nanjing, China). qRT‐PCR was performed on QuantStudio™ 7 Flex RT–PCR System (Applied Biosystems) using ChamQ Universal SYBR qPCR Master Mix (Vazyme Biotech, Nanjing, China). Three technical replicates were included. Relative expression levels were determined using 2^−ΔCT^ formula using *Actin* as internal reference.

### Barley stripe mosaic virus (BSMV) virus‐induced gene silencing (VIGS)

BSMV–VIGS analysis was conducted according to He *et al*. (2015). A 310 bp cDNA fragment of *Horvu_13821_1H01G536500* amplified from W1 was subcloned into BSMV:γ vector in an antisense orientation for BSMV:*HvBlp* construct (Table S1). Constructs were linearized and used for transformation as described by He *et al*. (2015). BSMV:*HvPDS* containing *HvPDS* was used as a positive control to validate the BSMV–VIGS system via infecting the second leaf of 10 day old W1 seedlings. At early milk stage, the W1 spikes were inoculated twice (7‐days interval) with BSMV:*HvBlp*. Spikes inoculated with the empty vector (BSMV:γ) served as mock treatment controls. Three biological replicates (each containing four spikes from different plants) were included for both mock and BSMV:*HvBlp* inoculation. After a 7 day of second inoculation, the specific husk/pericarp tissues were manually peeled off the embryo and endosperm (two spikes of each replicate) for RNA extraction to verify the knockdown of *HvBlp* by qRT–PCR. The VIGS experiments were repeated twice using different batches of plant materials.

### Whole‐genome long‐read sequencing

The yellow husk/pericarp mutant line yw1 was obtained from the following process: dry black‐coloured W1 seeds were treated with gamma rays at a dosage of 300 Gy. After the treatment, the seeds were continuously planted in the field (Hubei, China). A stably inherited mutant with yellow husk/pericarp trait was identified from the M_4_ generation, which was designated as yw1. Genomic DNA was extracted from the yw1 fresh leaves using DNeasy Plant Mini Kit (QIAGEN). The SMRT bell library was constructed using the Pacific Biosciences (PacBio) SMRT bell express template prep kit 2.0. Sequencing was carried out on the PacBio Revio platform of Benagen Technology Co., Ltd. (Wuhan, China). The SMRTlink v11.0 software was used to clean up the raw PacBio read data. Minimap2 (v2.17) was used to align the long‐reads to the Hor13821 genome, which was further sorted by samtools v1.9. Previously published short‐gun sequencing reads (SRR3655669—SRR3655670) for wild type W1 were downloaded from the ENV database (https://www.ebi.ac.uk/ena/browser/; Tan *et al*., 2020) and were mapped to the Hor13821 genome using bwa‐mem2 (v2.2.1) tool. All mapped reads were visualized using the Samplot tool (https://www.github.com/ryanlayer/samplot; Belyeu *et al*., 2021).

### Subcellular localization

The full‐length CDS of *HvBlp* without termination codon was fused into the pCAMBIA2300‐35S‐GFP vector via homologous recombination. Then, the pCAMBIA2300‐35S‐HvBlp‐GFP construct (HvBlp‐GFP) and pCAMBIA1300‐35S‐PM‐mCherry (PM: a plasma membrane marker) were co‐transformed into barley protoplasts and *Nicotiana benthamiana* leaves, as described previously (Jia *et al*., 2023; Xiao *et al*., 2022). The pCAMBIA2300‐35S‐GFP was used as a negative control (35S‐GFP). The GFP and mCherry fluorescence signals were captured using a confocal laser‐scanning microscope TCS SP8 CLSM (Leica, Germany).

### Evolutionary analysis

*HvBlp* homologues were identified using hmmscan in HMMER package (http://hmmer.org/), using domains PF01490 and PF00324 as queries. The annotated protein sequences for Hor13821 and other 41 species were retrieved from either Phytozome V12 (https://phytozome‐next.jgi.doe.gov/) or EnsemblPlant (https://plants.ensembl.org/index.html) databases. Sequences were aligned using MAFFT v7.505 with default parameters (Katoh and Standley, 2013). Maximum likelihood (ML) phylogenetic analysis was undertaken with IQ‐TREE (V1.6.12) under the best substitution model (JTT + F + G4; Nguyen *et al*., 2015). Neighbour joining (NJ) phylogenetic analysis was constructed by MEGA7 using *p*‐distance substitution model (Kumar *et al*., 2016). For each of phylogenetic analysis, 1000 bootstrap replicates were used to evaluate the reliability of the phylogenetic trees.

Natural selection pressure was analysed as described by Jia *et al*. (2023) using codeml in the PAML4.7 package (Yang, 2007). CDS sequence alignment of *Blp*s was performed using Muscle (Edgar, 2004). Branch pattern specification was implemented using Treeview1.6.6 (http://taxonomy.zoology.gla.ac.uk/rod/treeview.html). *P*‐value for Likelihood‐ratio tests was calculated using Graphpad software (https://www.graphpad.com/quickcalcs/PValue1.cfm).

### Metabolome analysis and data processing

A widely targeted metabolomics strategy was used to determine the metabolites in BINL (BS1 and BS3) and YNIL (YS1 and YS3) husk/pericarp tissues at S1 and S3 stages. The extraction, detection and quantification of metabolites in the samples were conducted by the Wuhan Metware Biotechnology Co., Ltd. (www.metware.cn). The metabolites were measured in three biological replicates using UPLC coupled with tandem mass spectrometry (MS/MS). All the metabolites were annotated by the MetWare database and quantified using multiple reaction monitoring. DAMs were identified based on the thresholds fold change (FC) ≥2 or FC ≤0.5, and variable importance in project (VIP) ≥1.

### RNA sequencing and co‐expression network analyses

Total RNA of barley and black‐coloured durum wheat DR638 were extracted using the TRNzol Universal Reagent (TIANGEN Biotech, China) for RNAseq analyses using a Nanopore PromethION and Illumina novaseq 6000 platforms. cDNA library construction and data analysis were performed for full‐length transcriptome sequencing and lncRNA sequencing as described by Yang *et al*. (2022) and Zong *et al*. (2021), respectively. Clean reads were aligned to the black barley Hor13821 genome (Jayakodi *et al*., 2020) using HISAT2 (Kim *et al*., 2019) tool. For durum wheat, *Triticum turgidum* reference genome was used. DESeq2 R package was used to identify the differentially expressed transcripts (DETs) and lncRNAs (DELs), with the cutoff parameters of fold‐change ≥2 and *P*‐value <0.05. KEGG enrichment analysis was performed using OmicShare tools (http://www.omicshare.com/tools). The Pearson correlation coefficient (PCC) among the DETs, DELs and DAMs were calculated using the Metware platform (https://cloud.metware.cn/). Then, the co‐expression network was visualized by Cytoscape software.

### Statistical analysis of data

All the experiments were conducted in triplicates. Data were analysed by SPSS 23.0, and significant differences were analysed using Student's *t*‐test in a one‐way analysis of variance (ANOVA) at *P* < 0.05.

## Funding

This work was supported by the Hubei Seed Industry High‐Quality Development Fund (HBZY2023A001)，Hubei Outstanding Youth Fund (2021CFA064), Hubei Agricultural Science and Technology Innovation Center Innovation Team Project (2021‐620‐000‐001‐01) and Leading Talent Program in Hubei Academy of Agricultural Sciences.

## Conflict of interest

The authors declare no competing interest.

## Author contributions

Y.X. and C.L. conceived the project and supervised this study. B.L., X.L., S.Z., Z.L., R.W., Y.G., W.Z. and C.J. performed the experiments. B.L. and Y.J. analysed the data and wrote the manuscript. Y.X. and C.L. revised and approved the manuscript.

## Supporting information


**Figure S1** The structural analysis of HvBlp and OsBh4 protein.

**Figure S2** Validation of Horvu_13821_1H01G536500 function via VIGS (repeat).

**Figure S3** Average sequencing depth and coverage in the barley genome.

**Figure S4** Genome‐wide identification and phylogenetic investigation of the AAT homologues.

**Table S1** Primers used in this study
**Table S2** The expression results of 11 candidate genes from our RNA sequencing data and public data
**Table S3** Statistics of the yw1 long‐read sequencing result
**Table S4** The information of 1072 metabolites
**Table S5** The details of 54 DAMs in cluster 8
**Table S6** The details of 37 core DAMs associated with HvBlp
**Table S7** The core DAMs, DETs and DELs co‐expressed with HvBlp

**Data S1** The profiles of ML tree construction.

**Data S2** The profiles of natural selection analysis.

## Data Availability

The RNA sequencing data in barley (full‐length transcriptome and lncRNA), wheat (DR638) and whole genome long reads sequencing (yw1) have been deposited to the National Center for Biotechnology Information (BioProject number: PRJNA951889).
