# Supplementary figures and images for: Transcriptional convergence after repeated duplication of an amino acid transporter gene leads to the independent emergence of the black husk/pericarp trait in barley and rice
Source: Plant Biotechnol J. 2023 Dec 20;22(5):1282–98. doi: 10.1111/pbi.14264 (PMC11022822; doi:10.1111/pbi.14264)

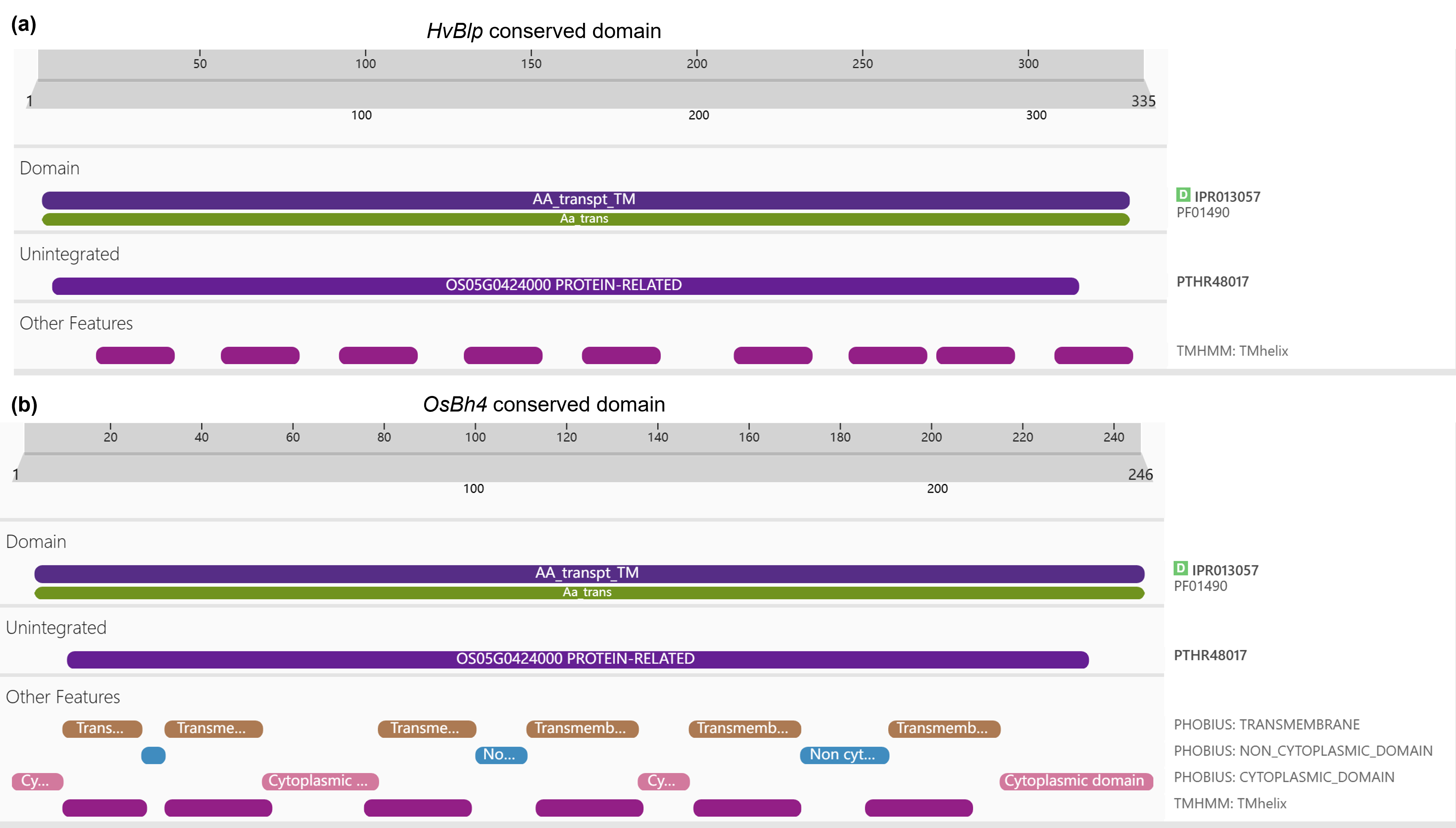

Supplement: Supplementary file 1 — Figure S1 The structural analysis of HvBlp and OsBh4 protein. [file PBI-22-1282-s001.tif]

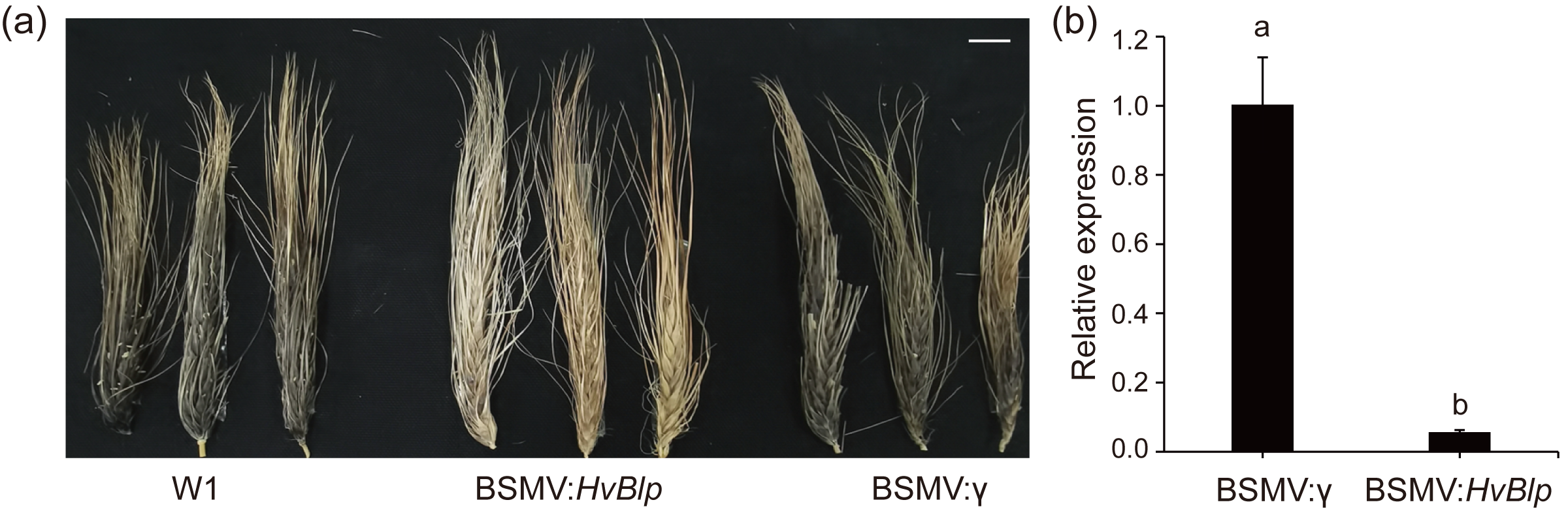

Supplement: Supplementary file 2 — Figure S2 Validation of Horvu_13821_1H01G536500 function via VIGS (repeat). [file PBI-22-1282-s003.tif]

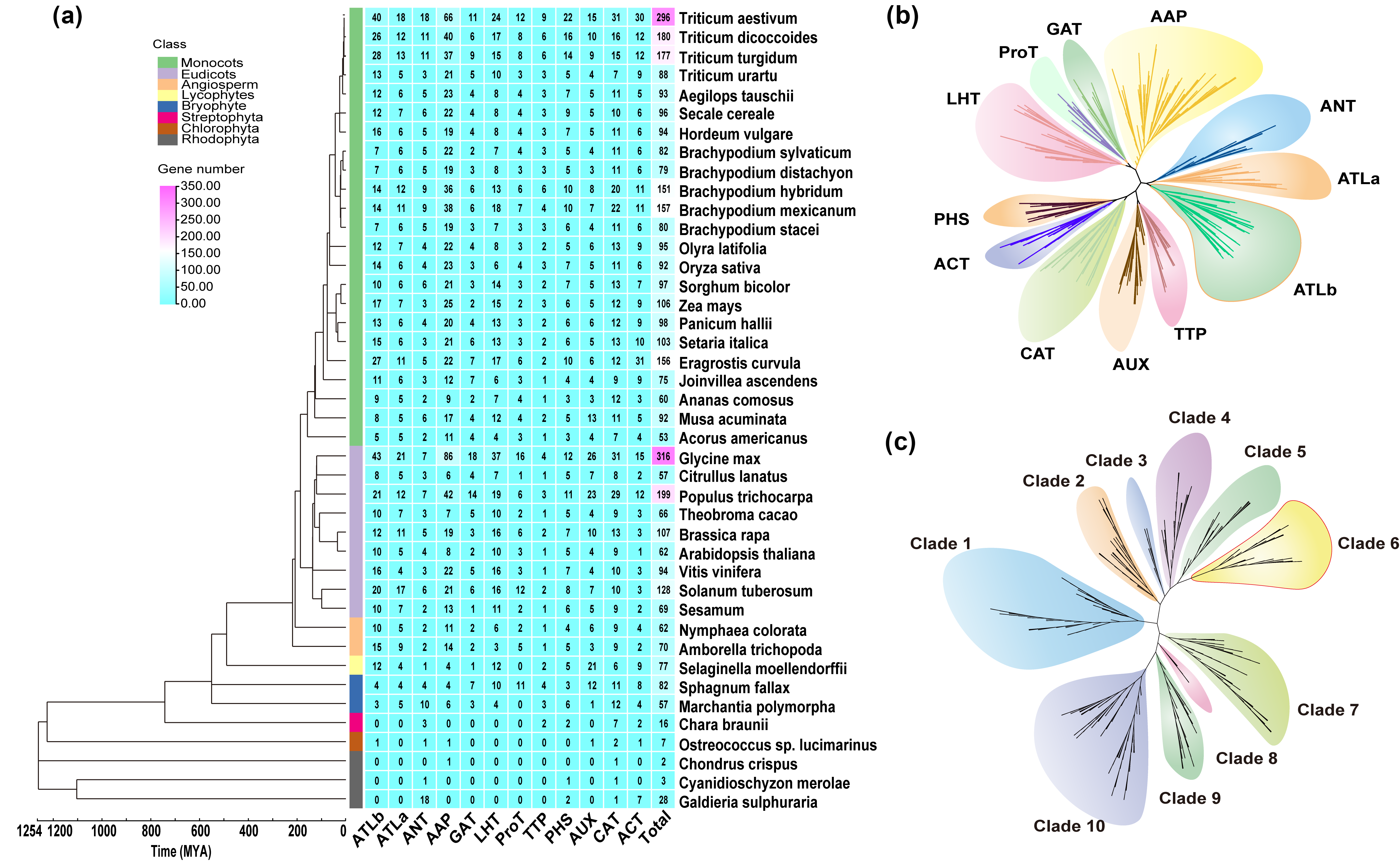

Supplement: Supplementary file 4 — Figure S4 Genome‐wide identification and phylogenetic investigation of the AAT homologues. [file PBI-22-1282-s004.tif]
